# Supplementary material for: Subcellular tracking reveals the location of dimethylsulfoniopropionate in microalgae and visualises its uptake by marine bacteria
Source: eLife. 2017 Apr 4;6:e23008. doi: 10.7554/eLife.23008 (PMC5380433; doi:10.7554/eLife.23008)
Supplement: Figure 1—source data 1. — DOI: http://dx.doi.org/10.7554/eLife.23008.004 [file elife-23008-fig1-data1.doc]

|  |  |  |  |  |
| --- | --- | --- | --- | --- |
| Compound | Mass (g) to make stock | Stock volume (mL) | Stock (mM) | Volume of stock/L of media (mL) |
| NaNO_3_ | 10.1988 | 40 | 3000 | 0.433 |
| Na_3_NTA | 10.284 | 40 | 1000 | 0.28 |
| H_3_BO_3_ | 1.2366 | 40 | 500 | 0.36 |
| KH_2_PO_4_ | 2.7218 | 40 | 500 | 0.296 |
| Na_2_EDTA | 0.74448 | 40 | 50 | 1.8 |
| NH_4_NO_3_ | 1.6008 | 40 | 500 | 0.03 |
| Thymine | 0.06305 | 40 | 12.5 | 0.256 |
| FeCl_3_·6 H_2_O | 0.5406 | 40 | 50 | 0.27 |
| Pyridoxine (HCl) | 0.08226 | 40 | 10 | 0.001 |
| Riboflavin | 0.00301 | 40 | 0.2 | 0.05 |
| Cyanocobalamin | 0.00542 | 40 | 0.1 | 0.05 |
|  |  |  |  |  |
