# Supplementary material for: Subcellular tracking reveals the location of dimethylsulfoniopropionate in microalgae and visualises its uptake by marine bacteria
Source: eLife. 2017 Apr 4;6:e23008. doi: 10.7554/eLife.23008 (PMC5380433; doi:10.7554/eLife.23008)
Supplement: Figure 2—source data 1. — Note, when the samples were collected, the Symbiodinium densities were not significantly different between the different treatments (T-Test, n = 8, t = 0.589, p=0.565). DOI: http://dx.doi.org/10.7554/eLife.23008.008 [file elife-23008-fig2-data1.doc]

|  |  |  |  |
| --- | --- | --- | --- |
| Treatment | Total DMSP (μmol/L)  (^1^H-NMR) | ^32^S-DMSP (%)  (LC-MS) | ^34^S-DMSP (%)  (LC-MS) |
| DMSP standard | NA | 93.62 | 6.39 |
| ^34^SO_4_^2-^ + *Pseudovibrio* sp. | 14.99 ± 0.47 | 54.33 | 45.68 |
| ^34^SO_4_^2-^ + *Escherichia coli* | 21.73 | 60.05 | 39.95 |
| ^34^SO_4_^2-^ + no bacteria | 22.55 | 56.84 | 43.16 |
| ^32^SO_4_^2-^ + no bacteria | 20.86 | 93.79 | 6.21 |
|  |  |  |  |
